# The Health‐Related Quality of Life Impact of the COVID‐19 Pandemic on People Living with Multiple Sclerosis and the General Population: A Comparative Study Utilizing the EQ‐5D‐5L with Psychosocial Bolt‐Ons

**DOI:** 10.1002/brb3.70210

**Published:** 2024-12-31

**Authors:** Glen J. Henson, Ingrid van der Mei, Bruce V. Taylor, Suzi B. Claflin, Andrew J. Palmer, Gang Chen, Julie A. Campbell

**Affiliations:** ^1^ Menzies Institute for Medical Research University of Tasmania Hobart Tasmania Australia; ^2^ Centre for Health Economics Monash University Caulfield East Victoria Australia

**Keywords:** COVID‐19, health state utility, multiple sclerosis, outcome measurement, quality of life

## Abstract

**Objectives:**

Studies have shown that people living with multiple sclerosis (PwMS) were substantially impacted by the COVID‐19 pandemic. However, no study has compared the overall health‐related quality of life impact of the COVID‐19 pandemic on PwMS and the general population. Differences would have implications for crises/pandemic management policies. This study aimed to compare the prevalence and health‐related quality of life impact of COVID‐19‐related adversity (such as deteriorations in mental or physical health) in PwMS and the general population.

**Methods:**

Cross‐sectional data were obtained from the How Is Your Life Australian general population study (comprising subsamples with and without chronic disease) and the Australian MS Longitudinal Study from August to October 2020. Health‐related quality of life was measured using health state utilities (HSUs; represented on a 0 [death] to 1 [full health] scale) generated by the EQ‐5D‐5L‐Psychosocial. COVID‐19‐related adversity was measured via specialized survey items. Descriptive and multivariable regression analyses were conducted.

**Results:**

A total of 1020 general population individuals and 1635 MS participants entered the study (mean age 52.4 and 58.4; female 52.4% and 80.2%, respectively). COVID‐19‐related adversity prevalence was higher among PwMS compared to the general population with and without chronic diseases (PR: 1.430 [CI: 1.153, 1.774] and PR: 1.90 [CI: 1.56, 2.32], respectively). However, the HSU impact of COVID‐19‐related adversity was not dependent on disease status (*p* > 0.20, test for interaction).

**Conclusion:**

This study found that PwMS were more likely to experience COVID‐19‐related adversity compared to the general population, though the health‐related quality‐of‐life impact was similar. This demonstrates that PwMS require additional support during national and global crises.

## 1 Introduction

### 1.1 Multiple Sclerosis in the Health Economics Context

Multiple sclerosis (MS) is a neurodegenerative disorder, currently without cure or certain etiology (McGinley, Goldschmidt, and Rae‐Grant 2021; Thompson, Baranzini, et al. 2018). Symptoms associated with MS are diverse and may include incoordination; motor, cognitive, sexual, bladder, and bowel dysfunction; sensory impairment; pain; and fatigue (Zhang et al. 2021). Consequently, people living with multiple sclerosis (PwMS) have varying experiences of the disease (Zhang et al. 2021). However, health‐related quality of life is often substantially impacted by the disease (J. A. Campbell, Ahmad, et al. 2023).

MS affected around 2.8 million people globally as of 2020 (Walton et al. 2020), and an estimated 33,335 Australians in 2021 (Ahmad et al. 2018; J. Campbell, van der Mei, Taylor, and Palmer 2023). Our comprehensive cost of illness study showed that the total, annual societal cost of MS in Australia was 2.45 billion Australian dollars in the 2021 calendar year (J. Campbell, van der Mei, Taylor, and Palmer 2023). The cost per person with MS per year was estimated at 73,457 Australian dollars (J. Campbell, van der Mei, Taylor, and Palmer 2023). MS‐related costs and global prevalence are predicted to increase at an accelerating rate (Walton et al. 2020; J. A. Campbell et al. 2020).

### 1.2 Health‐Related Quality of Life During the COVID‐19 Pandemic and Study Rationale

Several studies have contributed to our understanding of the effect of the COVID‐19 pandemic on health‐related quality of life. General population studies have frequently found significant impacts on psychological well‐being (Every‐Palmer et al. 2020; Grover et al. 2020; Huang and Zhao 2020; Karageorghis et al. 2021). Studies of chronic disease populations have identified limitations in access to health care as a principal vector through which the COVID‐19 pandemic has reduced quality of life. Regarding MS, most studies have focused on the mental health effects of the pandemic (Motolese et al. 2020; Costabile et al. 2021; Ramezani et al. 2021; Manacorda et al. 2021). While these studies have frequently concluded that PwMS were negatively impacted by the COVID‐19 pandemic, there is less conclusive evidence regarding whether PwMS were more affected than the general population. Upon examination of the literature, we found conflicting evidence presented across relevant studies, with some finding a difference (Yeni, Tulek, and Terzi. 2022) and others not (Motolese et al. 2020; Talaat et al. 2020; Altieri et al. 2022).

To our knowledge, only two studies have investigated how the COVID‐19 pandemic affected the broader health‐related quality of life of PwMS using a multiattribute utility instrument (namely the EQ‐5D‐5L [Ferreira et al. 2021] and the AQoL‐8D [Henson et al. 2024]). Using algorithms, these instruments convert patient‐reported responses into numerical scores, based on preestablished, country‐specific value sets (Drummond et al. 2015). These scores are known as health state utilities (HSUs) and are represented on a 0 (death) to 1 (full health) scale (Culyer 2014). HSU is an essential metric for clinical trials and cost‐utility studies, the latter of which informs resource allocation decisions (J. A. Campbell et al. 2016).

Importantly, no study has investigated whether the impact of the COVID‐19 pandemic differed between PwMS and the general population using HSUs. This is particularly significant as HSU is a uniquely holistic measure of health‐related quality of life (Drummond et al. 2015). A study using this measure may therefore contribute impactful evidence to the literature. Indeed, a recent systematic review, regarding differences in the psychological impact of the COVID‐19 pandemic on PwMS and healthy controls, called for further, related research (Altieri et al. 2022). This was due to the strong impact of the COVID‐19 pandemic on the general population's mental health and well‐being, which may have had an attenuating effect and led to the study's largely negative results. Moreover, we could identify no study that compared the impact of the COVID‐19 pandemic between PwMS and persons living with other chronic diseases generally.

### 1.3 Aims of the Study

This study aimed to determine if the impact of the COVID‐19 pandemic differed between PwMS and the general population (both with and without chronic diseases) through analysis of EQ‐5D‐5L‐Psychosocial HSUs. In doing so, it would contribute important information to a currently divided literature. If PwMS were more affected, this would indicate that they require additional resources to support their health‐related quality of life during pandemics and other isolating crises. Values determined by our study would be applicable in health economic simulation models that aim to evaluate the impact of and solutions to such crises.

## 2 Methods

### 2.1 Source of Study Participants

Participants living with MS (hereafter “PwMS sample”) were sourced from the Australian Multiple Sclerosis Longitudinal Study (AMSLS). The AMSLS is a representative, survey‐based cohort study that has been funded by MS Australia since 2002 (Taylor et al. 2013). Recruitment to the study is ongoing, and all study participants are required to provide informed consent before admittance. The AMSLS currently involves around 2600 active participants, with an estimated 96.0% of these participants diagnosed with MS per the McDonald criteria (Thompson, Banwell, et al. 2018). Ethics Approval for the AMSLS was received from the Tasmanian Health and Medical Human Research Ethics Committee (ethics approval number H0014183).

Participants from the Australian general population (hereafter “general population sample”) were drawn from the How is Your Life (HIYL) study. This study was conducted by Monash University's Centre for Health Economics. The HIYL study participants (aged 18 and over) were recruited through the global company Cint (www.cint.com) from its panel members. These participants were subsequently asked to complete an anonymous, online survey developed using Qualtrics (www.qualtrics.com). Recruitment for this study used a quota sampling method that was informed by the age and sex distributions in Australia. Ethics approval for the HIYL study was granted by the Monash University Human Research Ethics Committee (Project ID 8442).

### 2.2 Sources of Data

General population data were sourced from the HIYL online survey (conducted in September–October 2020). AMSLS data were sourced primarily from the 2020 Quality of Life survey (conducted August–October 2020), with additional data regarding education level and MS phenotype obtained from the 2018, 2019, and 2020 Disease Course surveys. Unique AMSLS research identifiers were used to link AMSLS data sources. All data used in this study were cross‐sectional, collected during a time of major lockdowns (isolation quarantines) in Australia (Henson et al. 2024) and in which moderate–high levels of distress were observed among the Australian population (Griffiths et al. 2022).

### 2.3 Measures

#### 2.3.1 Health‐Related Quality of Life and the EQ‐5D‐5L‐Psychosocial Instrument

Health‐related quality of life was measured using the EQ‐5D‐5L‐Psychosocial multiattribute utility instrument (Chen and Olsen 2020). Table S1 displays the nine items of EQ‐5D‐5L‐Psychosocial and each of their five levels. The EQ‐5D‐5L‐Psychosocial comprises the five items of the EQ‐5D‐5L (mobility, self‐care, usual activities, pain/discomfort, and anxiety/depression) and four bolted‐on, psychosocial items from the AQoL‐8D (vitality, sleep, personal relationships, and social isolation) (Chen and Olsen 2020). These psychosocial bolt‐ons were developed to improve the EQ‐5D‐5L's ability to capture mental and social aspects of health‐related quality of life (Chen and Olsen 2020); the EQ‐5D‐5L's relatively limited sensitivity to changes in psychosocial health has been discussed previously (Finch et al. 2017; Finch, Brazier, and Mukuria 2019; Yang et al. 2015). The added psychosocial questions were found to account for 45% of the explained variation in health‐related quality of life, as measured by the EuroQoL Visual Analogue Scale (measures health on a 0–100 numeric scale) (Chen and Olsen 2023).

As with other multiattribute utility instruments, the EQ‐5D‐5L‐Psychosocial represents health‐related quality of life on a 0 (death) to 1 (full health) scale. This instrument's Australian value set was developed by mapping AQoL‐8D utilities onto EQ‐5D‐5L‐Psychosocial health states (Chen and Olsen 2020). The new instrument has also been found to be interchangeable with the comprehensive, 35‐item AQoL‐8D while being substantially less burdensome (comprising nine items) (J. A. Campbell, Ahmad, et al. 2023). Additionally, the EQ‐5D‐5L‐Psychosocial instrument was recently validated, by our group, for use with MS and myalgic encephalomyelitis cohorts (J. A. Campbell, Ahmad, et al. 2023; Orji et al. 2023). Our study found that psychosocial domains of health are key contributors to the health‐related quality of life (and thus HSUs) of PwMS, especially the domains of sleep (Laslett et al. 2022; Braley and Boudreau 2016) and vitality (J. A. Campbell, Ahmad, et al. 2023).

Changes in HSUs can be evaluated using minimum important differences. A minimum important difference is the smallest change in HSU that is considered clinically meaningful and would support a change in disease management (Henson et al. 2022). In this study, we adopted a minimum important difference of 0.06 (Richardson et al. 2014). This minimal important difference has been used with the AQoL‐8D, which is interchangeable with EQ‐5D‐5L‐Psychosocial due to the instruments using the same value set. Similarly, EQ‐5D‐5L‐Psychosocial HSUs may also be evaluated against the AQoL‐8D population norm of 0.80 (Richardson et al. 2014).

#### 2.3.2 Measures of COVID‐19‐Related Adversity

For the sample of PwMS, COVID‐19‐related adversity (composed of perceived diminutions in health) was determined using a specialized COVID‐19 questionnaire, embedded in the AMSLS 2020 Quality of Life survey (see Figure S1). This questionnaire (Cronbach's alpha of 0.864) specifically required participants to rank the influence of the COVID‐19 pandemic, in seven quality of life domains, on 0–5 Likert scales. The questionnaire's content was informed by the domains included in the AQoL‐8D multiattribute utility instrument. Following examination of responses, this scale was simplified to 0 for no adverse effect (0–1), 1 for a minor adverse effect (2), and 2 for a major adverse effect (3–5) on the basis of differences in mean HSUs associated with the response categories (Henson et al. 2024).

In the HIYL study, COVID‐19‐related adversity was measured using a modified version of the Personal Wellbeing Index instrument, with responses to the overall life satisfaction item being extracted. Using this item, an ordinal variable was constructed, which indicated if an HIYL participant had been (1) adversely affected, (2) not affected, or (3) beneficially affected by the COVID‐19 pandemic.

The measures of COVID‐19‐related adversity were reduced to a dichotomous indicator variable (to facilitate interstudy data compatibility) for which a value of 1 indicated the presence of COVID‐19‐related adversity. A conservative approach was adopted in constructing this indicator, whereby members of the PwMS sample were considered to have reported COVID‐19‐related adversity if they reported a major adverse effect in at least one domain of health. Further detail regarding this is presented in Appendix A of the Supporting Information.

#### 2.3.3 Other Sociodemographic and Clinical Measures

Sociodemographic measures included age (stratified into the categories <35, 35–44, 45–54, 55–64, 65–74, >74), sex, education (secondary or less, occupational certificate or diploma, bachelor's degree, postgraduate degree), the Australian Bureau of Statistics’ Index of Relative Socioeconomic Advantage and Disadvantage (IRSAD; stratified by quartile [this variable also accounts for remoteness]), exposure to COVID‐19‐related lockdown (either metropolitan lockdown, rural lockdown, or neither), and Australian state of residence.

Clinical measures included MS‐related disability and MS phenotype (for the PwMS sample) and the presence of chronic diseases (for the general population sample). As in previous studies, MS‐related disability was mapped from the Patient Determined Disease Steps to the Expanded Disability Severity Scale (EDSS; 0–10) and categorized as no (0.0), mild (0.5–3.5), moderate (4.0–6.0), or severe disability (6.5–9.5) (J. A. Campbell, Ahmad, et al. 2023; Kobelt et al. 2006). Chronic diseases among the general population were grouped into the following classifications (which broadly reflect ICD‐10 Classifications/Codes) (World Health Organization 2015): psychological, musculoskeletal, respiratory, oncological, endocrinological, cardiovascular, gastrointestinal, neurological, sensory, and other. These data were used to classify two general population subgroups—those with and without chronic diseases. These subgroups were utilized in analyses.

Data were collected in functionally identical formats for both samples, except for COVID‐19‐related adversity (as discussed under Section 2.3.2). Differences in the characteristics of the samples’ participants are presented below. Importantly, adjustment for the above covariates, in regression, was utilized to facilitate intersample comparability.

### 2.4 Descriptive Analyses

Descriptive analyses involved the stratification of HSUs over key variables and sample/group membership. In all descriptive analyses, means and standard deviations were reported for continuous variables, while counts and proportions were adopted for categorical variables. Ordinal variables were summarized in both manners. All quantitative analyses were undertaken using Stata 17 (StataCorp, 2021).

### 2.5 Regression Analyses

Linear models, with HSU as the outcome variable, were used to identify differences in COVID‐19‐related adversity impacts and overall health‐related quality of life between the PwMS sample and the general population sample and subgroups. Despite the non‐normality of the HSU data (D'Agostino *K* ^2^ of 120.76, *p* < 0.01 [null hypothesis of normality]), model residuals were sufficiently gaussian (see Figure S2 for a related *Q*–*Q* plot and statistical test) that transformation of this dependent variable, prior to regressions, was not required to ensure valid statistical inferences, especially given our large sample size (*n* = 2656) (D'Agostino, Belanger, and D'Agostino 1990). In addition, log‐binomial regression models were estimated to investigate if COVID‐19‐related adversity was more prevalent in the PwMS sample compared to the general population sample and its subgroups. Log‐binomial models are analogous to logit models, except that they generate ratios of relative risk rather than odds.

Both univariable and multivariable regression models were estimated. Multivariable models were adjusted for all available covariates, including age, sex, employment status, education level, socioeconomic status by area, lockdown exposure (log‐binomial only), and reports of COVID‐19‐related adversity (linear only). Lockdown exposure and COVID‐19 adversity were not adjusted for simultaneously as they lie on the same causal pathway (as explained and evidenced elsewhere [Henson et al. 2024]), and COVID‐19 adversity was the subject of the log‐binomial regressions.

## 3 Results

### 3.1 Flow of Participants Into the Study

**FIGURE 1:**
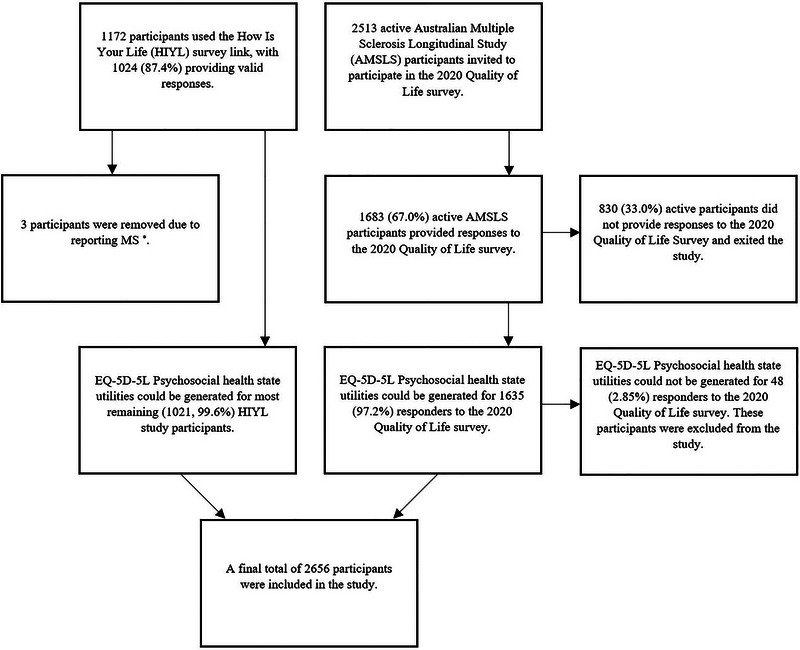
Flow of people living with multiple sclerosis and general population participants into the study. The asterisk indicates that HIYL participants self‐reporting MS were not transferred to the AMSLS dataset as their diagnoses could not be confirmed.

Figure 1 outlines the flow of participants into the study. Regarding the PwMS sample, 2513 active AMSLS participants were invited to participate in the AMSLS 2020 Quality of Life Survey. Of these, 1683 (67.0%) participants responded to the 2020 Quality of Life survey. EQ‐5D‐5L‐Psychosocial HSUs were generatable for 1635 of these participants (97.2%). Regarding the general population sample, 1172 people from the general population used the HIYL study survey link, with 1024 (87.4%) providing valid responses. Of these participants, three were excluded. This was as they reported having MS and their diagnoses could not be confirmed to be adherent to the McDonald criteria (Thompson, Banwell, et al. 2018).

### 3.2 Comparison of the MS and General Population Samples

Table 1 displays the sociodemographic and clinical characteristics of the MS and general population study samples. PwMS were on average 6 years older (58.4 vs. 52.4); however, this difference did not exceed 1 SD from the mean age of either sample. More importantly, the PwMS sample had a higher proportion of females than the general population sample (80.2% vs. 52.4%). This result is expected as MS disproportionately affects females at a 3:1 ratio (Thompson, Baranzini, et al. 2018; Orton et al. 2006). Additionally, participants in the PwMS sample were more likely to possess postgraduate qualifications (17.4% vs. 10.5%) and live in higher socioeconomic status areas (34.2% vs. 25.1%).

**TABLE 1:** Participant characteristics for the people living with multiple sclerosis (PwMS) and general population samples.

Variables	PwMS sample (*N* = 1635)	General population sample (*N* = 1021)
Age: Mean (SD)	58.4 (11.3)	52.4 (17.0)
Sex: *N* (%)		
Male	320 (19.6)	486 (47.6)
Female	1315 (80.4)	535 (52.4)
Education level: *N* (%)		
Secondary school or less	414 (25.3)	290 (28.4)
Occupation certificate or diploma	571 (34.9)	362 (35.5)
Bachelor's degree	354 (21.7)	259 (25.4)
Postgraduate degree	285 (17.4)	108 (10.5)
Unknown	11 (0.7)	2 (0.2)
Socioeconomic status by area: *N* (%)		
Well below average (first quantile)	187 (11.4)	182 (17.8)
Below average (second quantile)	256 (15.7)	179 (17.5)
Average (third quantile)	297 (18.2)	184 (18.0)
Above average (fourth quantile)	334 (20.4)	219 (21.5)
Well above average (fifth quantile)	560 (34.2)	256 (25.1)
Unknown	1 (0.1)	1 (0.1)
State: *N* (%)		
Australian Capital Territory	65 (4.0)	13 (1.3)
New South Wales	457 (28.0)	317 (31.1)
Northern Territory	2 (0.1)	8 (0.8)
Queensland	207 (12.7)	206 (20.2)
South Australia	165 (10.1)	77 (7.5)
Tasmania	94 (5.7)	18 (1.8)
Victoria	481 (29.4)	273 (26.7)
Western Australia	163 (10.0)	108 (10.6)
Unknown	1 (0.1)	1 (0.1)
COVID‐19‐related adversity: *N* (%)		
Reported adversity	700 (42.8)	300 (29.4)
Did not report adversity	922 (56.4)	721 (70.6)
Unknown	13 (0.8)	0 (0.0)
EDSS (PwMS sample): *N* (%)		
No disability	392 (24.0)	
Mild disability	341 (20.8)	
Moderate disability	588 (36.0)	
Major disability	303 (18.5)	
Unknown	11 (0.7)	
Phenotype (PwMS sample): *N* (%)		
Relapse Onset	1280 (78.3)	
Progressive Onset	219 (13.4)	
Unknown	136 (8.3)	
Chronic disease (general population sample): *N* (%)		
Reported chronic disease		602 (59.0)
Did not report chronic disease		364 (35.6)
Unknown		55 (5.4)
Psychiatric		250 (24.5)
Musculoskeletal		247 (24.2)
Respiratory		120 (11.8)
Oncological		28 (2.7)
Endocrinological		114 (11.2)
Cardiovascular		69 (6.8)
Gastrological		13 (1.2)
Neurological		40 (3.9)
Sensory		11 (1.1)
Other		14 (1.4)

*Note*: Diseases classified as “other” were used for categories that would have applied to <10 participants including autoimmune, dermatological, and hematological, among others.

Abbreviation: EDSS, Expanded Disability Severity Scale.

A total of 59.0% of the general population sample reported chronic diseases, and 54.5% of the PwMS sample had moderate or severe MS‐related disability. Among participants reporting chronic diseases, 24.5% and 24.2% reported psychiatric and musculoskeletal conditions, respectively. Also represented in the general population sample were oncological (11.8%) and endocrinological (11.2%) disorders. Other types of conditions were disclosed by less than 10.0% of the general population sample.

### 3.3 Distributions of EQ‐5D‐5L‐Psychosocial HSUs by Sample and Subgroup Membership

**FIGURE 2:**
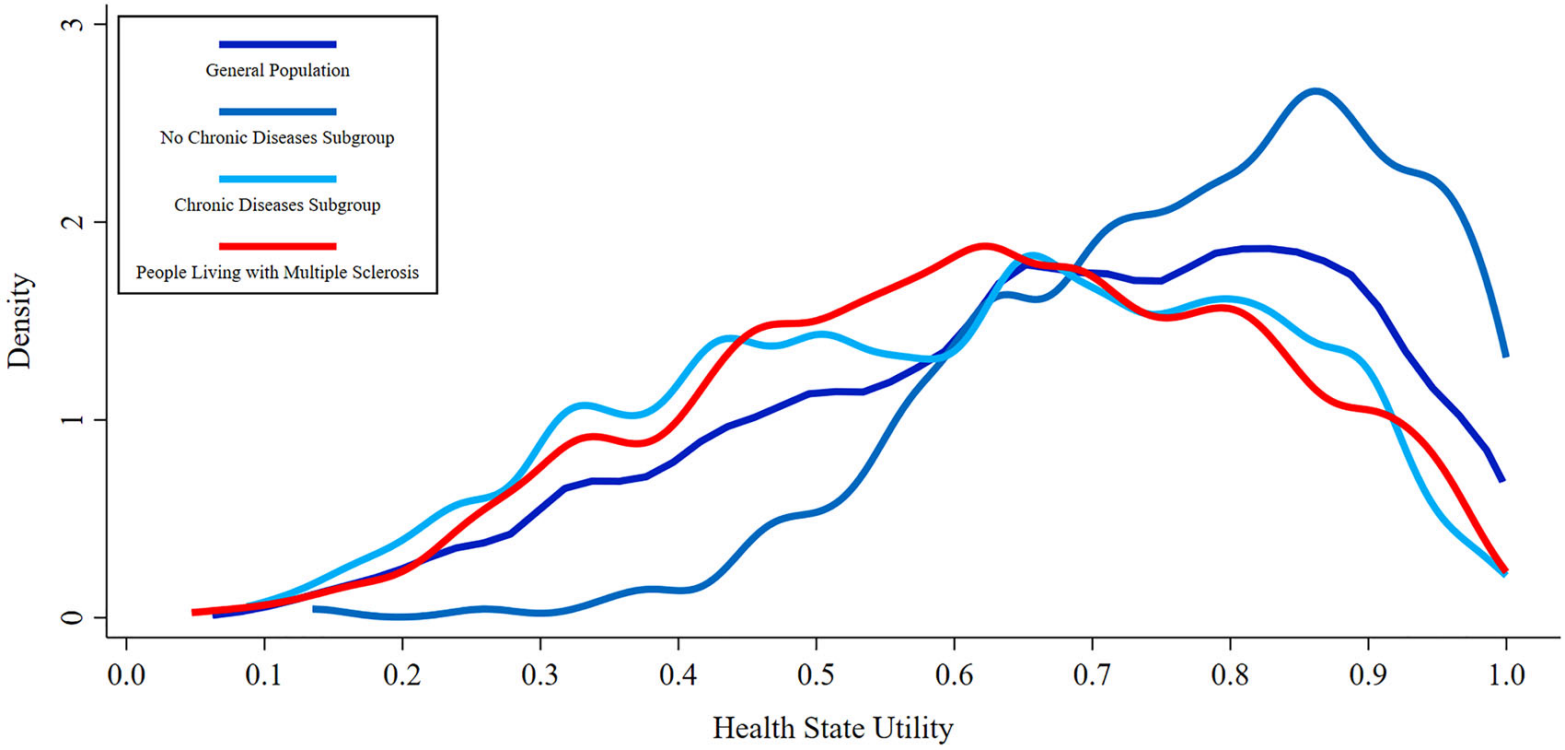
EQ‐5D‐5L‐Psychosocial health state utility kernel density estimates for all samples and subgroups.

Figure 2 contains a kernel density chart representing the distribution of each sample and subgroups’ HSUs, which are a measure of health‐related quality of life. The curve for the general population subgroup without chronic diseases shows that most members of this subgroup had HSUs above 0.700, with the highest density of HSUs around 0.850. The curves for the PwMS sample and the chronic disease subgroup illustrate that the distribution of HSUs for these groups is relatively similar, which implies comparability. Figure 2 is supported by Figures S3–S6, which provide individual histograms for each sample/subgroup's HSU distribution.

### 3.4 Stratifications of EQ‐5D‐5L‐Psychosocial Item Scores by Sample and Subgroup Membership

**FIGURE 3:**
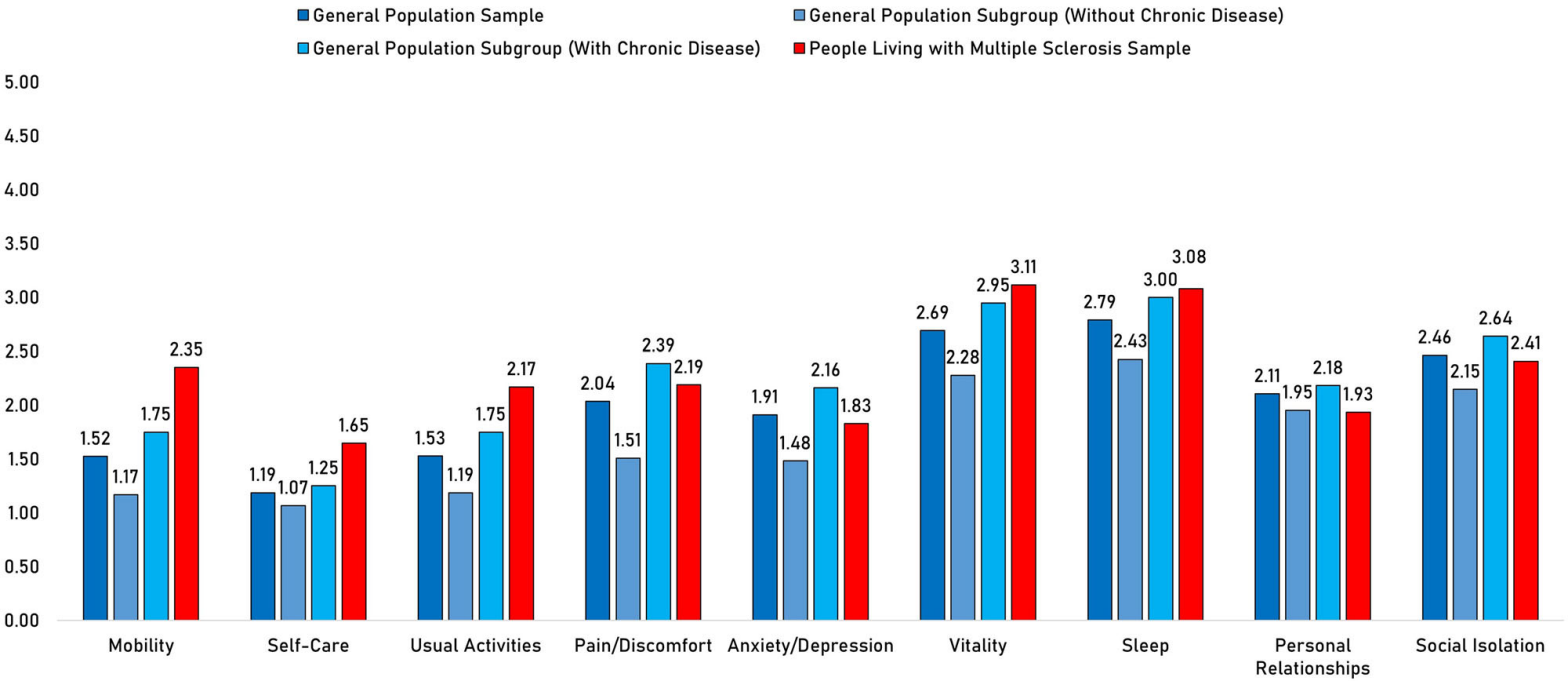
Column chart of mean EQ‐5D‐5L‐Psychosocial item scores for all samples and subgroups. Higher scores indicate greater disability.

Figure 3 (supported by Table S2) shows mean scores for the EQ‐5D‐5L‐Psychosocial items, stratified by sample and subgroup membership. Notably, PwMS scored worst, relative to the general population, in the domain of mobility, scoring 2.35 points on average (compared to 1.52 points, *p* < 0.01). Interestingly, all subgroups scored relatively poorly in the domains of vitality and sleep, compared to other domains.

### 3.5 Stratifications of Mean EQ‐5D‐5L‐Psychosocial HSUs by Key Measures

Table 2 demonstrates that mean HSUs for participants’ self‐reporting COVID‐19‐related adversity were lower in every sample and subgroup. Associated HSU decrements were consistently twice the 0.06 minimal important difference or greater. For example, the mean HSU of PwMS who reported COVID‐19‐related adversity was 0.512, compared to 0.671 for those who did not.

**TABLE 2:** Mean health state utilities for the multiple sclerosis and general population samples with and without stratification by COVID‐19‐related adversity.

Samples and subgroups	Health state utility: Mean (SD)		
	Overall	Not reporting COVID‐19‐related adversity	Reporting COVID‐19‐related adversity
People living with multiple sclerosis sample	0.616 (0.197)	0.671 (0.185)	0.512 (0.197)
General population sample	0.669 (0.204)	0.713 (0.191)	0.565 (0.199)
General population without chronic disease	0.776 (0.152)	0.803 (0.141)	0.684 (0.155)
General population with chronic disease	0.606 (0.206)	0.651 (0.197)	0.512 (0.197)

Table 3 displays mean HSUs stratified by disability severity, MS phenotypes, and chronic diseases (general population sample only). Across the categories of MS disability severity, mean HSU decreased from 0.791 to 0.501, a reduction almost five times the 0.06 minimum important difference. Table 3 also reveals that people living with progressive forms of MS had lower HSUs than people living with relapse onset MS (0.544 vs. 0.645), and among the chronic disease subgroup, persons with psychiatric conditions had the lowest HSUs (0.487, on average).

**TABLE 3:** Health state utilities for the multiple sclerosis and general population samples stratified by clinical classifications.

Sample and subgroup	Health state utility: Mean (SD)
People living with multiple sclerosis sample	
Disability severity (EDSS)	
No disability	0.791 (0.139)
Mild disability	0.661 (0.158)
Moderate disability	0.534 (0.167)
Severe disability	0.501 (0.501)
MS phenotype	
Relapse Onset	0.645 (0.197)
Progressive Onset	0.544 (0.205)
General population sample	
Chronic disease type	
Psychiatric	0.487 (0.188)
Musculoskeletal	0.593 (0.210)
Respiratory	0.600 (0.221)
Oncological	0.614 (0.185)
Endocrinology	0.620 (0.211)
Cardiovascular	0.590 (0.218)
Gastroenterology	0.622 (0.203)
Neurology	0.514 (0.234)
Sensory	0.643 (0.218)
Other	0.610 (0.203)

*Note*: MS‐related disability severity was classified with the Expanded Status Disability Scale (EDSS) as no disability (EDSS = 0), mild disability (EDSS = 0.5–3.5), moderate disability (EDSS = 4.0–6.0), and severe disability (EDSS = 6.5–9.5). Chronic diseases classified as “other” were appropriate for categories that would have applied to less than 10 participants. These diseases included autoimmune, dermatological, and hematological diseases, among others.

### 3.6 Associations Between Sample and Subgroup Membership and COVID‐19‐Related Adversity Impacts

Table 4 provides regression results regarding the HSU impact of COVID‐19‐related adversity. These results demonstrate that the association between reported COVID‐19‐related adversity and HSU was not substantially modified by sample/subgroup membership (*p* > 0.10 for all tests for interaction). Specifically, COVID‐19‐related adversity was associated with a 0.129‐point reduction in HSU, on average, for all participants.

**TABLE 4:** Univariable and multivariable linear regressions to determine the differences in the health state utility impact of COVID‐19‐related adversity.

Linear regressions with health state utility as the outcome				
	Univariable		Multivariable	
	Coefficient [95% CI]		Coefficient [95% CI]	
Two‐group comparison:				
General population	0.000	(Reference)	0.000	(Reference)
Multiple sclerosis	**−0.041**	**[−0.060, −0.022]**	**−0.050**	**[−0.070, −0.030]**
General population + COVID‐19‐related adversity	**−0.148**	**[−0.173, −0.122]**	**−0.150**	**[−0.175, −0.124]**
Multiple sclerosis + COVID‐19‐related adversity	**−0.168**	**[−0.187, −0.148]**	**−0.179**	**[−0.200, −0.158]**
Test for interaction between COVID‐19‐related adversity and multiple sclerosis	*p* = 0.200 for Wald test			

	Univariable		Multivariable	
	Coefficient [95% CI]		Coefficient [95% CI]	
Three‐group comparison:				
General population without chronic diseases	0.000	(Reference)	0.000	(Reference)
General population without chronic diseases + COVID‐19‐related adversity	**−0.119**	**[−0.164, −0.075]**	**−0.119**	**[−0.163, −0.075]**
General population with chronic diseases	**−0.152**	**[−0.180, −0.124]**	**−0.148**	**[−0.176, −0.120]**
General population with chronic diseases + COVID‐19‐related adversity	**−0.291**	**[−0.325, −0.257]**	**−0.287**	**[−0.321, −0.254]**
Multiple sclerosis	**−0.131**	**[−0.156, −0.107]**	**−0.143**	**[−0.169, −0.117]**
Multiple sclerosis + COVID‐19‐related adversity	**−0.257**	**[−0.284, −0.232]**	**−0.271**	**[−0.298, −0.245]**
Test for interaction between COVID‐19‐related adversity and general population with chronic disease	*p* = 0.463 for Wald test			
Test for interaction between COVID‐19‐related adversity and multiple sclerosis	*p* = 0.710 for Wald test			

*Note*: Tests for interaction utilized results obtained through multivariable regression. All multivariable regressions were adjusted for age, sex, education, and socioeconomic index. All bolded results were significant at an *α* = 0.001 level.

As presented in Table 5, examination of self‐reported COVID‐19‐related adversity as an outcome revealed that PwMS were 1.496 (*p* < 0.001) times more likely to report COVID‐19‐related adversity compared to the total general population sample, and 1.901 (*p* < 0.001) times more likely than the general population subgroup without chronic diseases. Additionally, members of the general population with chronic diseases were 1.430 times (*p* < 0.001) more likely to report COVID‐19‐related adversity than those without chronic diseases.

**TABLE 5:** Univariable and multivariable log‐binomial regressions to determine the associations between sample and subgroup membership and the prevalence of COVID‐19‐related adversity.

Log‐binomial regressions with COVID‐19‐related adversity as the outcome			
		Univariable	Multivariable
	Absolute proportion reporting COVID‐19‐related adversity	Prevalence ratio (95% CI)	Prevalence ratio (95% CI)
Two‐group comparison			
General population	29.38%	0.000 (Reference)	0.000 (Reference)
Multiple sclerosis	43.97%	**1.496 (1.341, 1.670)**	**1.485 (1.315, 1.678)**
Three‐group comparison			
General population without chronic diseases	23.90%	0.000 (Reference)	0.000 (Reference)
General population with chronic diseases	32.06%	**1.342 (1.080, 1.667)**	**1.430 (1.153, 1.774)**
Multiple sclerosis	43.97%	**1.839 (1.519, 2.227)**	**1.901 (1.557, 2.321)**

*Note*: All multivariable regressions were adjusted for age, sex, education, socioeconomic index, and lockdown exposure. All bolded results were significant at an *α* = 0.001 level.

## 4 Discussion

We found that the prevalence of self‐reported COVID‐19‐related adversity, defined as a self‐perceived reduction in well‐being associated with the pandemic, was greater among people living with chronic diseases, especially MS. This adversity was associated with clinically meaningful reductions in health‐related quality of life. The magnitude of this effect was not modified by having a chronic disease, including MS.

### 4.1 Differences in the Health‐Related Quality‐of‐Life Impact of the COVID‐19 Pandemic Between PwMS and the General Population

We identified that a greater proportion of participants living with MS reported COVID‐19‐related adversity compared to the general population. As noted above, this adversity was associated with clinically meaningful reductions in health‐related quality of life. Our study also found that sample/subgroup membership did not modify the association between COVID‐19‐related adversity and HSU.

A review of the quality of life literature identified a study that used the MS‐specific, Multiple Sclerosis Quality of Life‐54 instrument to measure health‐related quality of life, and which supported our findings (Yeni, Tulek, and Terzi. 2022). It concluded that PwMS were more impacted by the COVID‐19 pandemic versus healthy controls. Interestingly, studies utilizing generic, symptom‐specific instruments yielded opposing results, finding no difference in the impact of the pandemic on PwMS. One such study used the Depression Anxiety Stress Score‐21 (Talaat et al. 2020), and another applied the Beck Depression Inventory‐II and the Generalised Anxiety Disease‐7, among other instruments (Motolese et al. 2020).

### 4.2 Potential Causes of Worse Pandemic Outcomes for PwMS and Suggested Interventions

Reviews of relevant literature indicated that emotional health was a key route through which the COVID‐19 pandemic may have impacted the health‐related quality of life of PwMS (Altieri et al. 2022; Zarghami et al. 2022). Specifically, PwMS may have felt particularly isolated during the pandemic (Henson et al. 2024; J. A. Campbell, van der Mei, Taylor, et al. 2023) and experienced greater anxiety regarding potential COVID‐19 infection (due to the use of immunosuppressive therapies) (Manacorda et al. 2021; Learmonth et al. 2022). The COVID‐19 pandemic's impact on self‐care routines may also have been a major contributor to reduced health‐related quality of life for PwMS. In particular, the pandemic led to worsened diet and exercise routines (Marck et al. 2021), as well as feelings of abandonment resulting from reduced access to self‐care and healthcare resources (Manacorda et al. 2021).

Interventions have been proposed in the literature that would counteract the negative impacts of the COVID‐19 pandemic—and other isolating crises—on PwMS. In particular, remote delivery of health services (i.e., telehealth, which may include meetings with healthcare professionals, counselling, or trainer‐led exercise routines) was recommended (Learmonth et al. 2022). Other studies have indicated that engendering effective coping strategies may represent a proactive method for improving crisis outcomes among PwMS (Morris‐Bankole and Ho 2021; Pakenham 2006). These studies also suggested that active coping strategies are likely to be the most efficacious.

### 4.3 Strengths and Limitations

The greatest strengths of this study were the large samples from which data were obtained. These samples were diverse in terms of sociodemographic and clinical factors. Therefore, study results may be generalisable to a variety of populations. Moreover, the general population subgroup with chronic diseases had an HSU distribution similar to the PwMS sample. This implies comparability between the two groups and thus internal validity in this respect.

Regarding limitations, the EQ‐5D‐5L‐Psychosocial does not yet have a tailored minimum important difference or population norm. However, this was ameliorated by the similarities between the output of the EQ‐5D‐5L‐Psychosocial and the AQoL‐8D, which are interchangeable and use the same value sets (J. A. Campbell, Ahmad, et al. 2023). Additionally, COVID‐19‐related adversity data were extracted for each sample using different items. Our conservative approach to determining the presence of COVID‐19‐related adversity in PwMS (supported by Appendix A), and our choice of a simple, dichotomous indicator to represent COVID‐19‐related adversity, acknowledged this limitation and were intended to limit its influence on study results.

## 5 Conclusions

The prevalence of COVID‐19‐related adversity was much greater among people living with chronic diseases, especially MS, than among the general population without chronic diseases. Reductions in health‐related quality of life, associated with COVID‐19‐related adversity, were consistently both statistically significant and clinically meaningful. Given our findings, it is apparent that during isolating crises, such as pandemics, the management of health‐related quality of life among PwMS may require greater per capita investment, compared to that which is necessary for either the general population or people living with other chronic diseases.

## Author Contributions

**Glen J. Henson**: conceptualization, data curation, formal analysis, investigation, methodology, writing–original draft, validation. **Ingrid van der Mei**: data curation, methodology, resources, writing–review and editing, supervision. **Bruce V. Taylor**: methodology, writing–review and editing, supervision. **Suzi B. Claflin**: writing–review and editing, supervision. **Andrew J. Palmer**: writing–review and editing. **Gang Chen**: conceptualization, data curation, investigation, methodology, resources, writing–review and editing, validation. **Julie A. Campbell**: conceptualization, data curation, formal analysis, funding acquisition, investigation, methodology, resources, validation, supervision.

## Conflicts of Interest

The authors declare no conflicts of interest.

### Peer Review

The peer review history for this article is available at https://publons.com/publon/10.1002/brb3.70210

## Supporting information



Supplementary Materials

## Data Availability

Data is available, upon reasonable request, from the corresponding author.
