# Supplementary material for: The Health‐Related Quality of Life Impact of the COVID‐19 Pandemic on People Living with Multiple Sclerosis and the General Population: A Comparative Study Utilizing the EQ‐5D‐5L with Psychosocial Bolt‐Ons
Source: Brain Behav. 2024 Dec 31;15(1):e70210. doi: 10.1002/brb3.70210 (PMC11688049; doi:10.1002/brb3.70210)
Supplement: Supplementary file 1 — Supplementary Materials [file BRB3-15-e70210-s001.docx]

**The health-related quality of life impact of the COVID-19 pandemic on people living with multiple sclerosis and the general population: a comparative study**

**Supplementary Materials**

| **Title** | **Description** | **Page** |
| --- | --- | --- |
| *Supplementary Tables* |  |  |
| Supplementary Table 1 | EQ-5D-5L Psychosocial Items | 2 |
| Supplementary Table 2 | Proportions of Samples and Subgroups Selecting each Level of the EQ-5D-5L-Psychosocial’s Items | 3 |
| *Supplementary Figures* |  |  |
| Supplementary Figure 1 | Image of the COVID-19 questionnaire embedded in the Australian Multiple Sclerosis Longitudinal Study 2020 Quality of Life survey | 4 |
| Supplementary Figure 2 | Q-Q plot for linear regression model residuals | 5 |
| Supplementary Figure 3 | Histogram of EQ-5D-5L-Psychosocial health states utilities for the general population sample | 6 |
| Supplementary Figure 4 | Histogram of EQ-5D-5L-Psychosocial health states utilities for the general population subgroup without chronic disease | 7 |
| Supplementary Figure 5 | Histogram of EQ-5D-5L-Psychosocial health states utilities for the general population subgroup with chronic disease | 8 |
| Supplementary Figure 6 | Histogram of EQ-5D-5L-Psychosocial health states utilities for the multiple sclerosis sample | 9 |
| *Appendix* |  |  |
| Appendix A (One and Only) | Choice of method for the construction of a dichotomous COVID-19-related adversity indicator to be applied in the multiple sclerosis sample | 10 |

**Table of Contents**

**Supplementary Tables**

**Supplementary Table 1:** EQ-5D-5L Psychosocial Items

| *Items measuring physical health* | ***1. Mobility (EQ-5D-5L Item 1)***  (1) I have no problems in walking about  (2) I have slight problems in walking about  (3) I have moderate problems in walking about  (4) I have severe problems in walking about  (5) I am unable to walk |
| --- | --- |
|  | ***2. Self-Care (EQ-5D-5L Item 2)***  (1) I have no problems washing or dressing myself  (2) I have slight problems washing or dressing myself  (3) I have moderate problems washing or dressing myself  (4) I have severe problems washing or dressing myself  (5) I am unable to wash or dress myself |
|  | ***3. Usual Activities (EQ-5D-5L Item 3)***  (1) I have no problems doing my usual activities  (2) I have slight problems doing my usual activities  (3) I have moderate problems doing my usual activities  (4) I have severe problems doing my usual activities  (5) I am unable to do my usual activities |
|  | ***4. Pain/Discomfort (EQ-5D-5L Item 4)***  (1) I have no pain or discomfort  (2) I have slight pain or discomfort  (3) I have moderate pain or discomfort  (4) I have severe pain or discomfort  (5) I have extreme pain or discomfort |
|  |  |
| *Items measuring psychosocial health* ^A^ | ***5. Anxiety/Depression (EQ-5D-5L Item 5)***  (1) I am not anxious or depressed  (2) I am slightly anxious or depressed  (3) I am moderately anxious or depressed  (4) I am severely anxious or depressed  (5) I am extremely anxious or depressed |
|  | ***6. Vitality (AQoL-8D Item 1)***  (1) I am always full of energy  (2) I am usually full of energy  (3) I am occasionally full of energy  (4) I am usually tired and lacking energy  (5) I am always tired and lacking energy |
|  | ***7. Sleep (AQoL-8D Item 12)***  (1) I have no trouble sleeping  (2) I have trouble sleeping rarely  (3) I have trouble sleeping occasionally  (4) I have trouble sleeping frequently  (5) I have trouble sleeping always |
|  | ***8. Personal Relationships (AQoL-8D Item 10)***  (1) I am very satisfied with my close relationships  (2) I am somewhat satisfied with my close relationships  (3) I am neither satisfied nor dissatisfied with my close relationships  (4) I am somewhat dissatisfied with my close relationships  (5) I am very dissatisfied with my close relationships |
|  | ***9. Social Isolation (AQoL-8D Item 31)***  (1) I never feel isolated from my community  (2) I rarely feel isolated from my community  (3) I sometimes feel isolated from my community  (4) I often feel isolated from my community  (5) I always feel isolated from my community |

^A^ Items six through nine are based on but not identical to items included in the AQoL-8D.

| Item Levels ^B^ | ***General Population Sample*** | ***General Population Subgroup (Without Chronic Diseases)*** | ***General Population***  ***Subgroup (With Chronic Diseases)*** | ***People Living with Multiple Sclerosis Sample*** |
| --- | --- | --- | --- | --- |
|  | *Mobility* | | | |
| 1 | 67.1 (685) | 87.6 (319) | 54.2 (326) | 34.6 (580) |
| 2 | 17.7 (181) | 8.5 (31) | 23.4 (141) | 24.6 (412) |
| 3 | 11.4 (116) | 3.6 (13) | 16.3 (98) | 20.4 (341) |
| 4 | 3.5 (36) | 0.0 (0) | 5.8 (35) | 10.6 (177) |
| 5 | 0.3 (3) | 0.3 (1) | 0.3 (2) | 9.9 (165) |
|  | *Self-Care* | | | |
| 1 | 87.5 (893) | 95.3 (347) | 83.1 (500) | 62.0 (1036) |
| 2 | 7.5 (77) | 2.7 (10) | 10.5 (63) | 20.8 (348) |
| 3 | 4.0 (41) | 1.7 (6) | 5.1 (31) | 10.4 (174) |
| 4 | 0.8 (8) | 0.3 (1) | 1.0 (6) | 3.2 (54) |
| 5 | 0.2 (2) | 0.0 (0) | 0.3 (2) | 3.5 (59) |
|  | *Usual Activities* | | | |
| 1 | 66.5 (679) | 86.8 (316) | 53.3 (321) | 33.6 (562) |
| 2 | 19.6 (200) | 8.5 (31) | 26.3 (158) | 30.1 (503) |
| 3 | 10.2 (104) | 3.6 (13) | 14.9 (90) | 24.7 (413) |
| 4 | 2.3 (23) | 0.8 (3) | 3.3 (20) | 8.4 (141) |
| 5 | 1.5 (15) | 0.3 (1) | 2.2 (13) | 3.2 (54) |
|  | *Pain/Discomfort* | | | |
| 1 | 34.5 (352) | 56.3 (205) | 19.9 (120) | 25.9 (432) |
| 2 | 38.4 (392) | 36.8 (134) | 39.4 (237) | 38.7 (647) |
| 3 | 18.4 (187) | 6.6 (24) | 26.3 (158) | 26.9 (450) |
| 4 | 6.8 (69) | 0.3 (1) | 11.1 (67) | 7.6 (127) |
| 5 | 2.1 (21) | 0.0 (0) | 3.3 (20) | 0.9 (15) |
|  | *Anxiety/Depression* | | | |
| 1 | 46.6 (476) | 62.9 (229) | 37.7 (227) | 43.2 (721) |
| 2 | 28.2 (288) | 28.0 (102) | 27.4 (165) | 36.1 (603) |
| 3 | 16.5 (168) | 8.0 (29) | 21.3 (128) | 16.8 (281) |
| 4 | 5.2 (53) | 0.3 (1) | 8.3 (50) | 2.8 (47) |
| 5 | 3.5 (36) | 0.8 (3) | 5.3 (32) | 1.1 (19) |
|  | *Vitality* | | | |
| 1 | 7.3 (75) | 11.0 (40) | 4.3 (26) | 2.0 (34) |
| 2 | 40.2 (410) | 55.8 (203) | 31.7 (191) | 25.2 (422) |
| 3 | 33.5 (342) | 28.6 (104) | 35.9 (216) | 38.8 (649) |
| 4 | 14.4 (147) | 3.6 (13) | 21.3 (128) | 27.0 (453) |
| 5 | 4.6 (47) | 1.1 (4) | 6.8 (41) | 7.0 (117) |
|  | *Sleep* | | | |
| 1 | 14.2 (145) | 18.7 (68) | 11.8 (71) | 6.8 (114) |
| 2 | 25.3 (258) | 33.2 (121) | 21.1 (127) | 21.8 (365) |
| 3 | 37.2 (380) | 36.1 (135) | 36.4 (219) | 37.6 (629) |
| 4 | 14.2 (145) | 8.8 (32) | 17.1 (103) | 23.8 (398) |
| 5 | 9.1 (93) | 2.2 (8) | 13.6 (82) | 10.1 (169) |
|  | *Personal Relationships* | | | |
| 1 | 31.2 (318) | 34.0 (124) | 30.6 (184) | 35.3 (592) |
| 2 | 41.0 (429) | 45.3 (165) | 38.9 (234) | 42.7 (717) |
| 3 | 17.5 (179) | 14.6 (53) | 17.4 (105) | 16.3 (273) |
| 4 | 7.9 (81) | 4.7 (17) | 10.0 (60) | 4.9 (82) |
| 5 | 2.4 (24) | 1.4 (5) | 3.1 (19) | 0.8 (14) |
|  | *Social Isolation* | | | |
| 1 | 23.2 (237) | 29.9 (109) | 19.5 (118) | 24.5 (410) |
| 2 | 29.0 (296) | 35.2 (128) | 26.0 (155) | 29.9 (501) |
| 3 | 31.0 (316) | 26.4 (96) | 32.9 (198) | 29.6 (496) |
| 4 | 12.0 (122) | 7.1 (26) | 14.7 (89) | 13.1 (220) |
| 5 | 4.9 (50) | 1.4 (5) | 6.9 (42) | 3.0 (50) |

**Supplementary Table 2:** Proportions of Samples and Subgroups Selecting each Level of the EQ-5D-5L-Psychosocial’s Items

^B^ The item levels represented in this column are displayed in full in Table 1.

Data is presented in the format: percentage (number).

**Supplementary Figures**

**
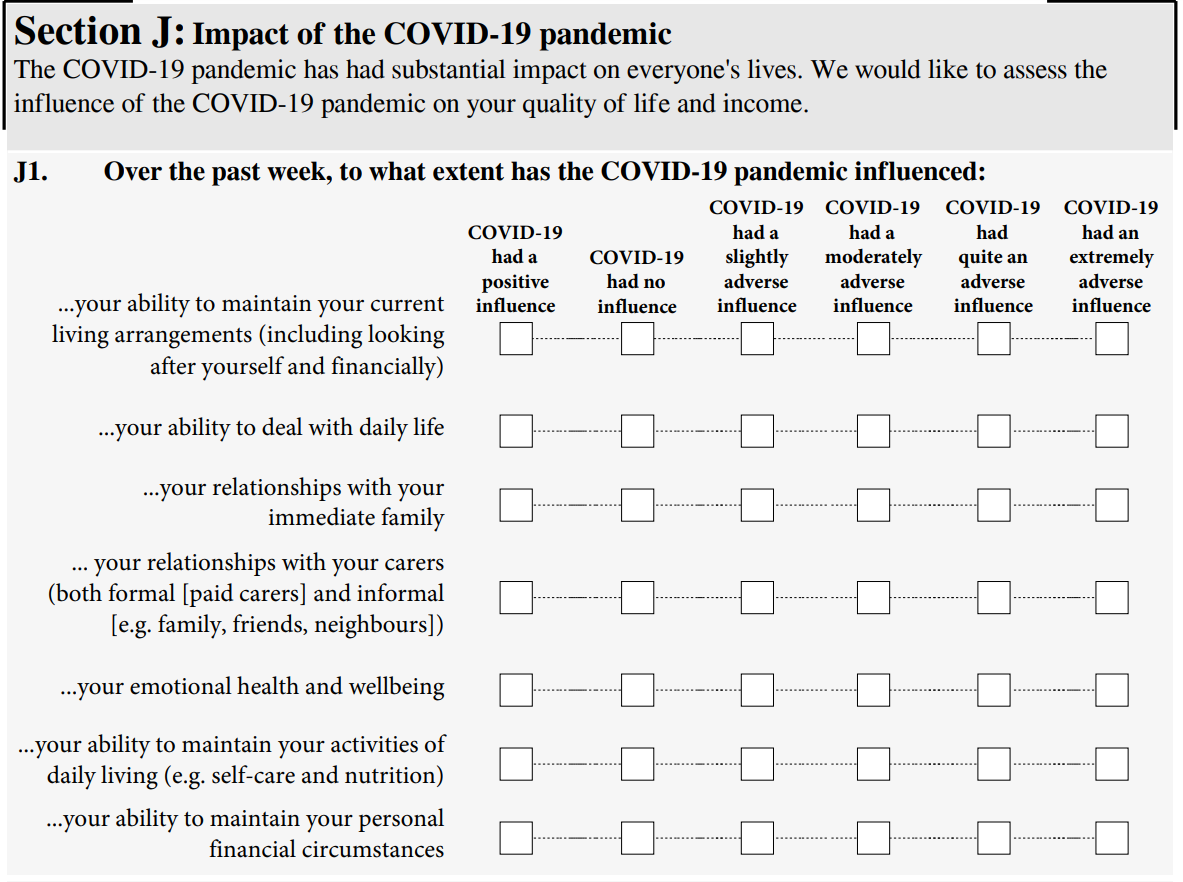
Supplementary Figure 1:** Image of the COVID-19 questionnaire embedded in the Australian Multiple Sclerosis Longitudinal Study 2020 Quality of Life survey

In order, the questions measure adversity in the health domains of independent living, coping with life, familial relationships, carer relationships, emotional wellbeing, self-care, and financial security.

**Supplementary Figure 2:** Q-Q Plot for linear model residuals
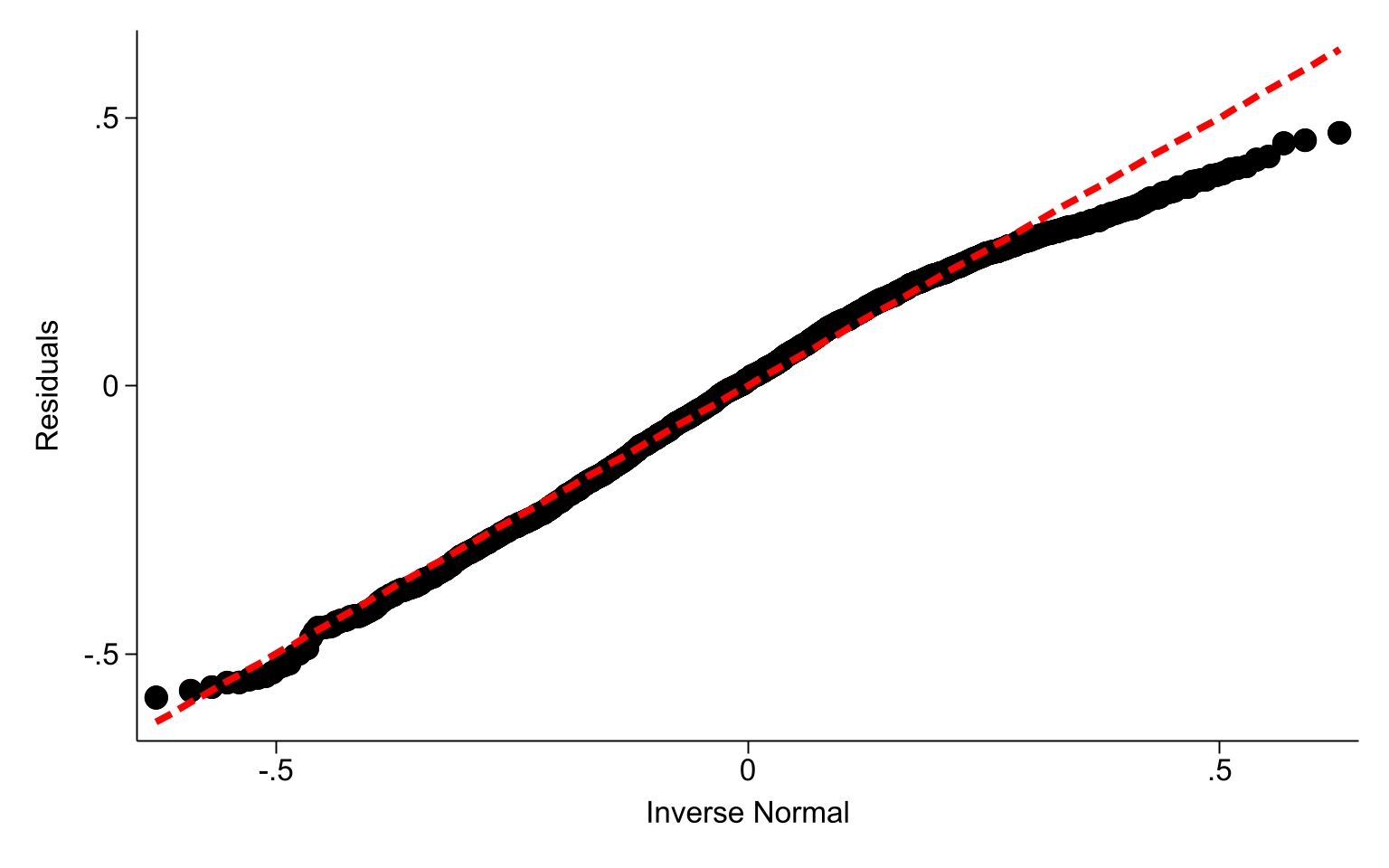


Substantial deviation of the plotted coordinates from the red 45 degree line, which extends from the origin, would be indicative of problematic non-normality in the distribution of model residuals. Such deviation is not observable in the above plot. Note that given the size of our sample (*n=2656*) statistical tests for normality would be overpowered and therefore inappropriate. For example, the D’Agostino K^2^ associated with the residuals is 61.18, resulting in the rejection of the null hypothesis of normality at an α level of 0.0001.

**
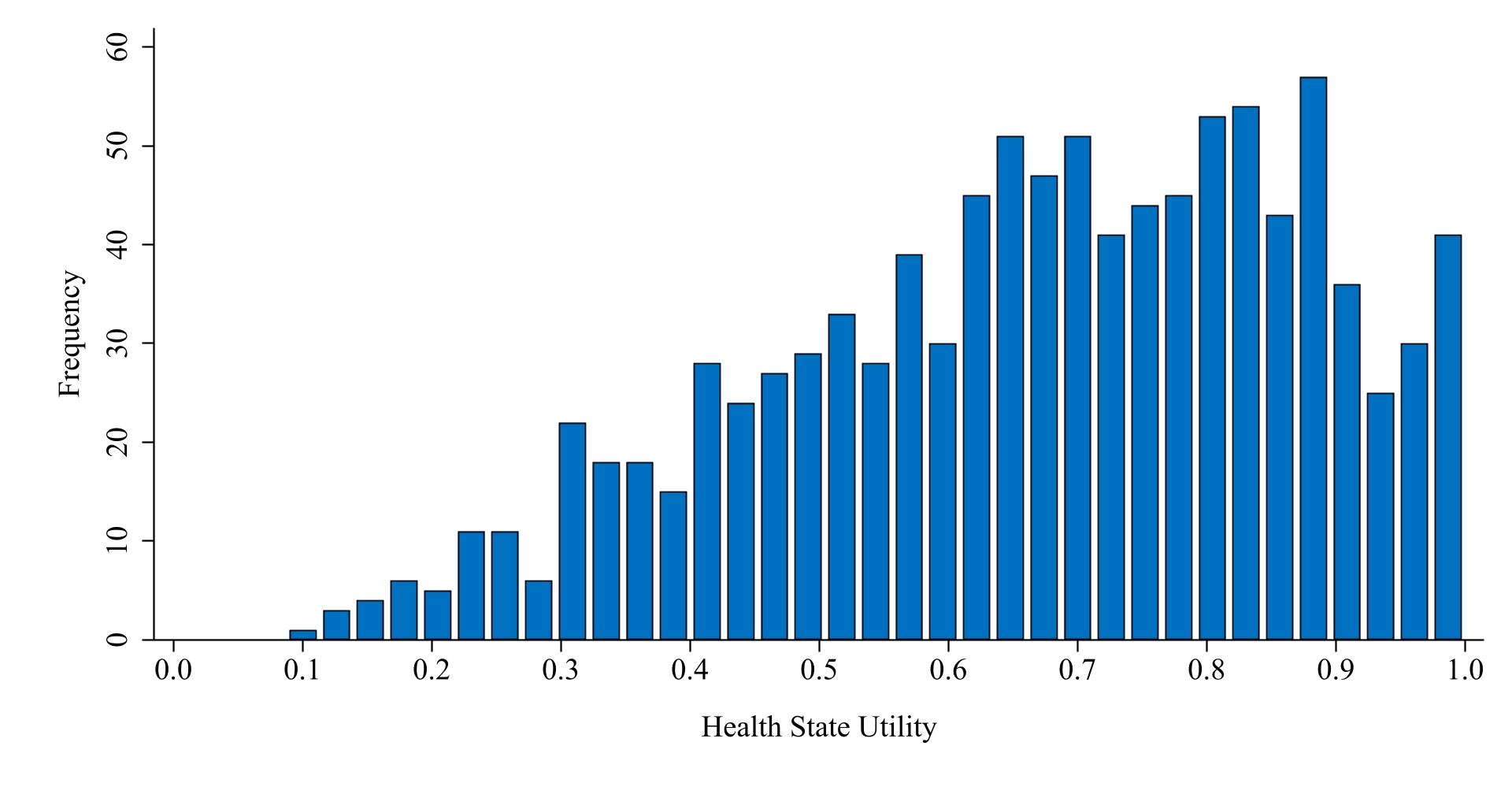
Supplementary Figure 2:** Histogram of EQ-5D-5L-Psychosocial health states utilities for the general population sample

**Mean = 0.669**

**Median = 0.692**

**SD = 0.205**

***
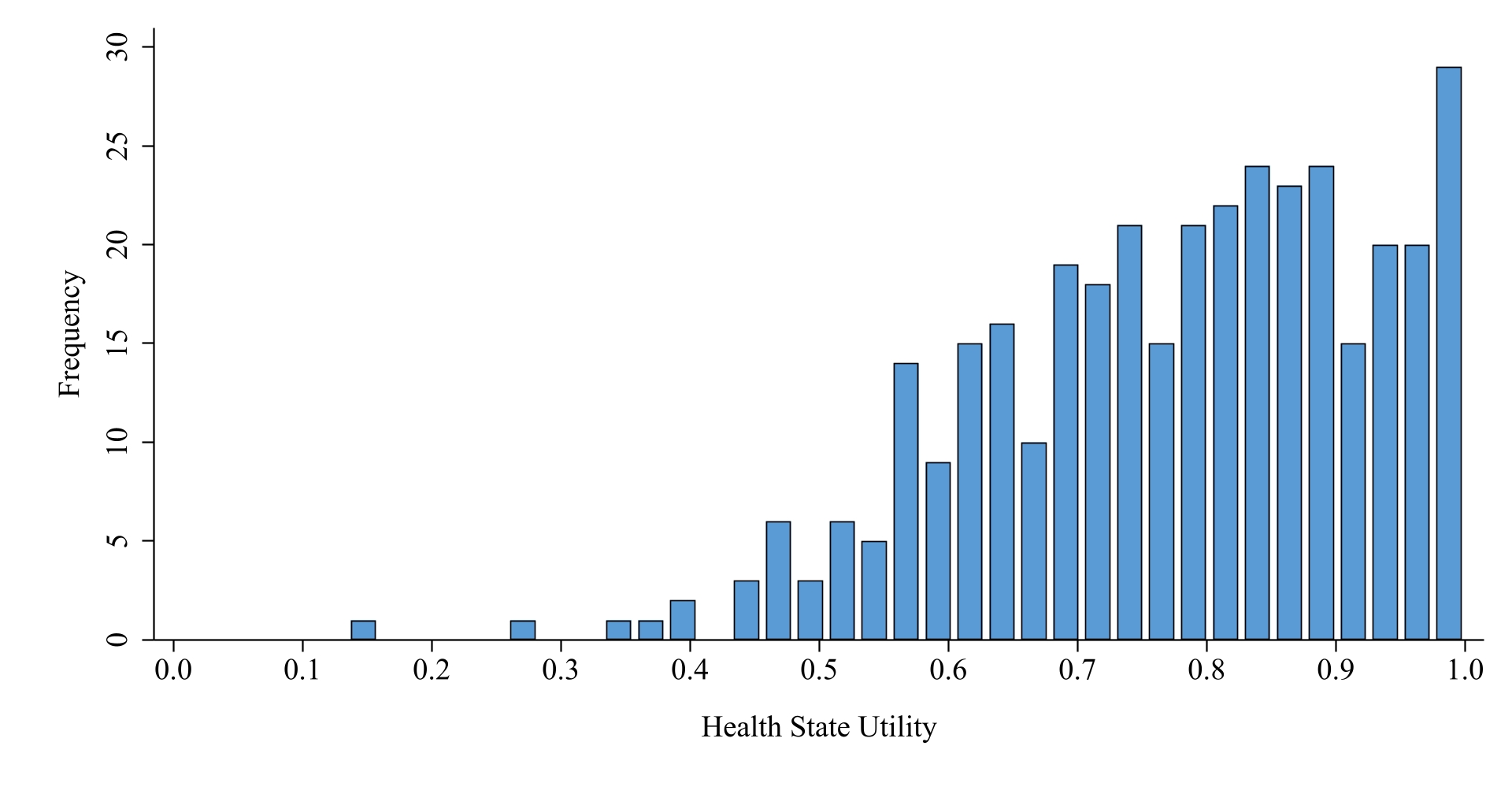
*Supplementary Figure 3:** Histogram of EQ-5D-5L-Psychosocial health states utilities for the general population subgroup without chronic disease

**Mean = 0.774**

**Median = 0.795**

**SD = 0.153**

***
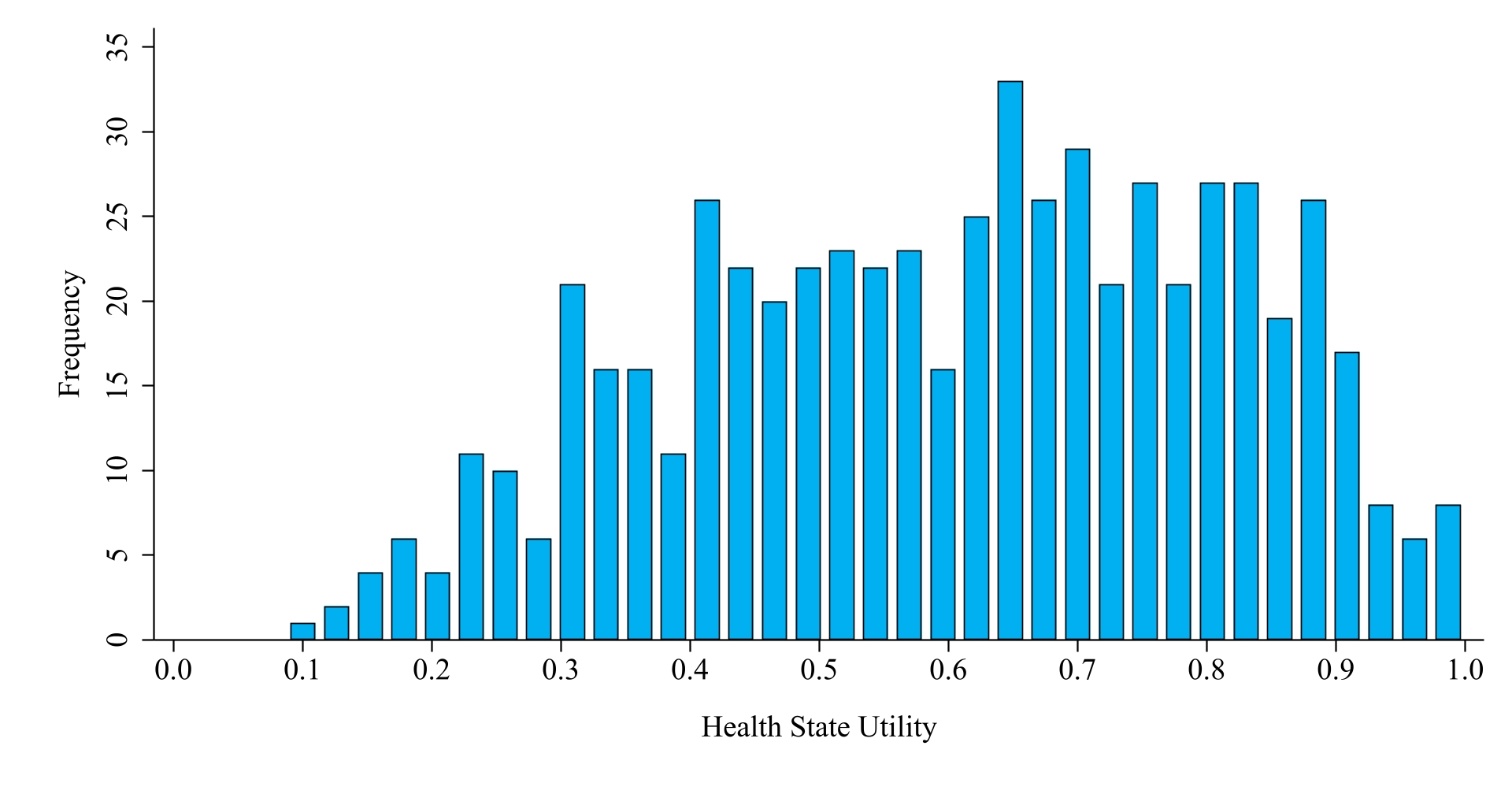
*Supplementary Figure 4:** Histogram of EQ-5D-5L-Psychosocial health states utilities for the general population subgroup with chronic disease

**Mean = 0.607**

**Median = 0.630**

**SD = 0.207**

***
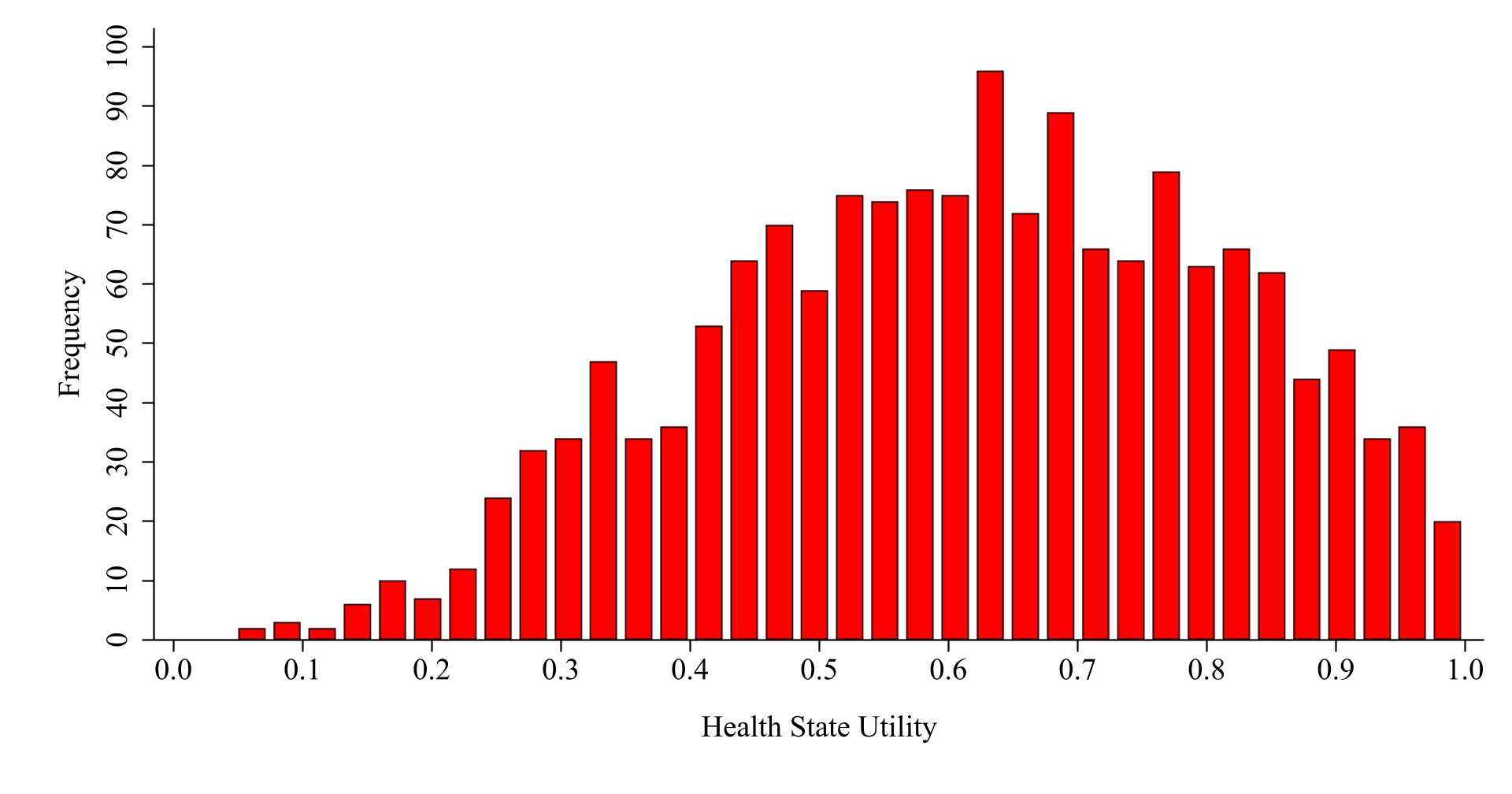
*Supplementary Figure 5:** Histogram of EQ-5D-5L-Psychosocial health states utilities for the people living with multiple sclerosis sample

**Mean = 0.616**

**Median = 0.625**

**SD = 0.197**

**Appendix A**

***Choice of method for the construction of a dichotomous COVID-19-related adversity indicator to be applied in the multiple sclerosis sample***

*1. Method selection criterion*

Regression showed that the effect of COVID-19 adversity on health state utility (HSU) was not modified by disability severity in the people living with multiple sclerosis (MS) sample. An illustrative example is provided below, which interacts reported COVID-19-related emotional adversity with Expanded Disability Status Scale (EDSS) category as a measure of MS-related disability.

**Appendix Table 1:** Interaction between disability severity and COVID-19-related emotional adversity

|  | Coefficient | (95% CI) |
| --- | --- | --- |
| Mild disability and minor emotional adversity | 0.00 | (-0.05, 0.05) |
| Mild disability and major emotional adversity | 0.02 | (-0.04, 0.08) |
| Moderate disability and minor emotional adversity | 0.03 | (-0.01, 0.08) |
| Moderate disability and major emotional adversity | 0.00 | (-0.05, 0.05) |
| Severe disability and minor emotional adversity | -0.02 | (-0.08, 0.04) |
| Severe disability and major emotional adversity | -0.01 | (-0.07, 0.06) |

The reference categories are no disability and no COVID-19-related emotional adversity.

Similarly, no interaction was identified between COVID-19-related adversity and reports of chronic disease in the How Is Your Life Study sample (see Table 5). As such, any dichotomous, COVID-19-adversity variable constructed for the MS sample was expected to not modify the association between EDSS and HSU. This was the selection criterion.

*2. Methods for analysis*

Three possible methods of constructing a dichotomous COVID-19-related adversity indicator for the people living with MS sample were considered: (1) construct an unweighted, arithmetic average of domain-specific COVID-19-related adversity scores (recalling that domain-specific scores took the forms 0 [no adversity], 1 [minor adversity], and 2 [major adversity]) and categorise scores equal to greater than 0.5714 (i.e., reporting at least minor adversity across a majority of domains) as representing sufficient COVID-19-related adversity; (2) regard participants reporting major adversity in any domain as having experienced sufficient COVID-19-related adversity; and (3) regard participants reporting any COVID-19-related adversity as having experienced sufficient COVID-19-related adversity.

*3. Evaluation of methods*

The first stage of analysis involved examining the relative prevalence of COVID-19-related adversity in the people living with MS sample using the different measures. Methods (1) and (2) produced similar prevalence results (1014, 38.2%; 1000, 37.7%), while method (3) gave a substantially higher prevalence (1504, 56.6%). In contrast, no measure modified the association between EDSS and HSU (data not shown).

Methods (1) and (2) performed similarly in descriptive and regression analyses, being largely interchangeable. However, when using method (3) MS was identified as modifying the relationship between COVID-19-related adversity and HSU (0.048, CI: 0.014, 0.081). Given this, method (3) failed to achieve the selection criterion. In deciding between the remaining methods, method (2) was chosen over method (1). This is because it was simpler and more conservative.
